# Identification and Characterization of an Exonic Duplication in *PALB2* in a Man with Synchronous Breast and Prostate Cancer

**DOI:** 10.3390/ijms23020667

**Published:** 2022-01-08

**Authors:** Ahmed Bouras, Cyril Lafaye, Melanie Leone, Zine-Eddine Kherraf, Tanguy Martin-Denavit, Sandra Fert-Ferrer, Alain Calender, Nadia Boutry-Kryza

**Affiliations:** 1 Genetic and Cancer Medical Laboratory HCL-CLB, Hospices Civils de Lyon, 69008 Lyon, France; cyril.lafaye@lyon.unicancer.fr (C.L.); melanie.leone@lyon.unicancer.fr (M.L.); alain.calender@chu-lyon.fr (A.C.); nadia.boutry-kryza@lyon.unicancer.fr (N.B.-K.); 2 Institute for Advanced Biosciences, University Grenoble Alpes, INSERM, CNRS, 38000 Grenoble, France; zekherraf@chu-grenoble.fr; 3 UM GI-DPI, University Hospital Grenoble Alpes, 38000 Grenoble, France; 4 Genetics Departement, Hospices Civils de Lyon, 69500 Bron, France; tanguy.martin-denavit@labo-alpigene.fr; 5 Center for Medical Genetics, Alpigène, 69007 Lyon, France; 6 Centre Hospitalier Métropole Savoie, Genetics Departement, 73011 Chambery, France; Sandra.Fert-Ferrer@ch-metropole-savoie.fr

**Keywords:** breast cancer, prostate cancer, *PALB2*, exon duplication

## Abstract

*PALB2* (partner and localizer of *BRCA2*), as indicated by its name, is a *BRCA2*-interacting protein that plays an important role in homologous recombination (HR) and DNA double-strand break (DSB) repair. While pathogenic variants of *PALB2* have been well proven to confer an increased risk of breast cancer, data on its involvement in prostate cancer (PrC) have not been clearly demonstrated. We investigated, using targeted next generation sequencing (NGS), a 59-year-old Caucasian man who developed synchronous breast and prostate cancers. This genetic investigation allowed to identify an intragenic germline heterozygous duplication in *PALB2*, implicating intronic repetitive sequences spanning exon 11. This variant was confirmed by multiplex ligation probe amplification (MLPA), and genomic breakpoints have been identified and characterized at the nucleotide level (c.3114-811_3202-1756dup) using an approach based on walking PCR, long range PCR, and Sanger sequencing. RT-PCR using mRNA extracted from lymphocytes and followed by Sanger sequencing revealed a tandem duplication r.3114_3201dup; p.(Gly1068Glufs * 14). This duplication results in the synthesis of a truncated, and most-likely, non-functional protein. These findings expand the phenotypic spectrum of *PALB2* variants and may improve the yield of genetic diagnoses in this field.

## 1. Introduction

Breast cancer remains the most frequent malignancy affecting women. This cancer is treated successfully in ~70–80% of cases diagnosed during the early stage [1]. Among all cases, 10% of patients seem to have a hereditary form of the disease (HBC). Genetic variants found in the breast cancer associated genes, *BRCA1* (OMIM #113705) and *BRCA2* (OMIM #612555), are considered as the most well-known breast cancer susceptibility factors. The lifetime cancer risk in carriers of pathogenic variants in these genes is estimated at 60–80% [2]. Therefore, routine genetic testing in HBC should focus on these two genes. Other novel candidate genes have subsequently been reported in this indication after negative testing for *BRCA1-2* variants in several HBC cases such as *CDH1*, *PALB2*, *PTEN*, and *TP53* [3,4]. Regarding this highly heterogeneous genetic basis, genetic screening of HBC is shifting from testing BRCA genes to broader panel testing.

Male breast cancer (MBC) is a rare disease corresponding to less than 1% of breast cancers and about 0.5% of the malignancies in men in Western countries. The genetic basis underlying MBC predisposition is strong, and therefore, it is highly recommended to test affected men for high-penetrance germline pathogenic variants (PVs) in breast cancer susceptibility genes, regardless of family history of cancer [5].

*PALB2* (partner and localizer of *BRCA2*, *OMIM* #610355) is localized on chromosome 16 and encodes for a *BRCA2*-interacting protein that serves as the molecular scaffold in the formation of the *BRCA1–PALB2–BRCA2* complex. It is therefore crucial for homologous recombination and DNA double-strand break (DSB) repair [6]. As *BRCA2*, germinal homozygous loss of function (LoF) variants of *PALB2* lead also to Fanconi anemia [7,8], whereas heterozygous LoF variants have been associated with HBC and pancreatic cancer [9,10,11]. However, while pathogenic mutations in *PALB2* have been recently associated with an increased risk of male breast cancer (MBC) [5], studies on its involvement in prostate cancer have given conflicting results. No previous study that we know has demonstrated statistically significant associations of *PALB2* with PrC risk [11,12,13,14].

Here, we report the identification, using next-generation sequencing of a *PALB2* complex variant (Alu-mediated exonic duplication) and its characterization and validation at the DNA and the RNA levels, of a man with synchronous prostate and breast cancer.

## 2. Results

### 2.1. Case Presentation and NGS Analysis

The proband—a 59-year-old man presented to the clinical genetics service at Hospices Civils de Lyon following the diagnosis of unilateral breast cancer (ER+, PR−, HER2−). A few months prior, the patient had undergone a prostatectomy following the discovery of prostate cancer. Post-operative staging and grading exposed a tumor without nodal metastasis and negative surgical margins (pT2c, N0, M0, R0) and a Gleason score of 3 + 4 = 7 (Prognostic Grade Group 2).

In his family history, we find a nephew who died at the age of 25 from a cancer of unknown origin. The rest of the family history was not suggestive of an inherited cancer predisposition syndrome (Figure S1).

In order to identify any genetic variants that could explain the phenotype, the proband was analyzed using the CE-IVD Hereditary Cancer Solution (HCS) assay by SOPHiA Genetics. The results revealed *PALB2* exon 11 duplication.

### 2.2. MLPA Analysis and Breakpoint Characterization

**Figure 1:**
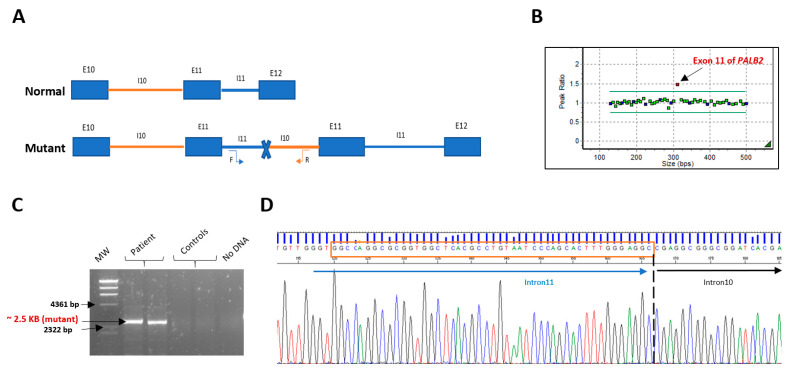
*PALB2* Exon 11 duplication testing and breakpoint identification. (**A**) Schematic representation of the *PALB2* normal and mutant alleles, with the duplicated region. The location and orientation of the primers used for specific duplication PCRs are indicated by vertical arrows. (**B**) *PALB2* Exon 11 duplication confirmation by MLPA. (**C**) Long-range PCR. A specific band of approximately 2.5 kb was detected in the proband’s blood DNA, absent in control samples. (**D**) Electropherogram showing the breakpoint sequence (forward). Tandem duplication site (TDS) is boxed in orange.

To confirm the identified duplication, MLPA was performed on the genomic DNA of the proband using the SALSA MLPA P260 *PALB2-RAD50-RAD51C-RAD51D* probe mix (MRC Holland, Amsterdam, The Netherlands). The results confirmed the presence of this germline duplication in the heterozygous state (Figure 1A).

**Figure 2:**
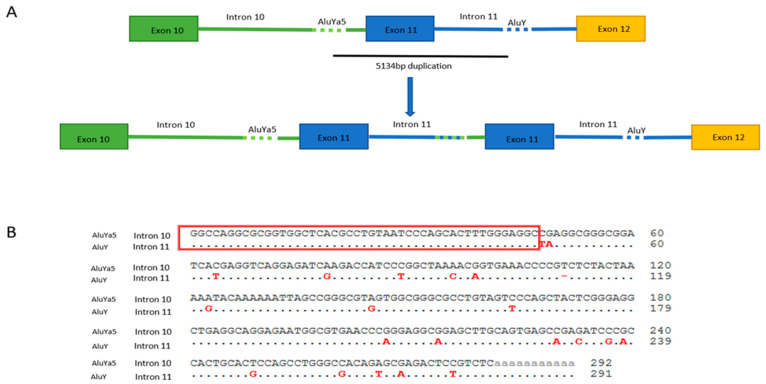
An Alu-mediated duplication mechanism appears to be responsible for exon 11 duplication. (**A**) Sequence analysis of the LR-PCR product allowed us to identify the duplication conjunctions that are located between c.3114-811 and 3202-1756. The location and orientation of the primers used for specific-duplication PCRs are indicated by vertical arrows. (**B**) Sequence alignment of the two Alu elements involved in this *PALB2* rearrangement showing that these two elements are highly homologous.

To better characterize the *PALB2* Exon 11 duplication, we confirmed this rearrangement by amplifying the genomic sequence spanning the exon 11. This assay confirmed the presence of an approximately 2.5 Kb specific PCR product in the proband, which was absent in normal control samples. Sanger sequencing analysis of the PCR amplicon revealed a 47-nucleotide element overlapping the two introns, resulting in a duplicated region of 5134 bp and allowing the accurate description of the duplication at DNA level (c.3114-811_3202-1756dup) (Figure 1). According to the Repeat-Masker software analysis, two Alu elements oriented in the same direction are present in the regions flanking the genomic breakpoint, one in intron 10 (*AluYa*5) and the other in intron 11 (*Alu*Y). Sequence alignment showed that these two elements are highly homologous (Figure 2).

### 2.3. RNA Analysis

**Figure 3:**
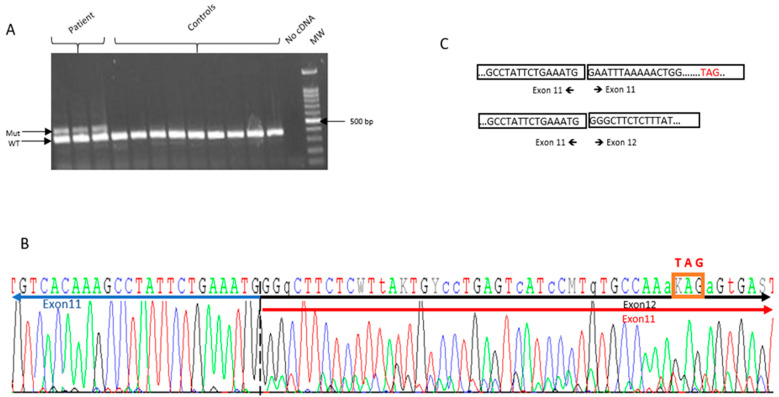
*PALB2* Exon 11 duplication disrupts normal splicing and leads to frameshift. (**A**) Agarose gel electrophoresis of the RT-PCR performed with mRNA obtained from the patient and non-carrier controls. An extra band was observed in the patient of about 474 bp, while only the wild-type band was observed in controls (388 bp). (**B**) Sanger sequencing of the two alleles of RT-PCR product of the sample from proband: the insertion leads to a new stop codon as indicated by the red box. (**C**) Schematic representation of the two transcripts observed in the proband sample.

The impact of *PALB2* exon 11 duplication at the RNA level was evaluated by performing an RT-PCR on the proband’s RNA by a single primer set to amplify the *PALB2* cDNA sequence flanking exon 11. We obtained two bands with different sizes after gel electrophoresis: a band at the expected size (388 bp) of the amplified fragment from the wild-type allele and a long band of about 474 bp. Sanger sequencing analysis of the long fragment evidenced that the duplication is in tandem. This abnormal transcript leads to a frameshift and a premature stop codon, thus confirming the deleterious impact of the identified variants on the protein functional structure (Figure 3).

## 3. Discussion

In the present study, we identified, by NGS, an exonic duplication occurring in the *PALB2* gene in a man who developed synchronous breast and prostate cancers at 59. The identified duplication was verified and validated by MLPA, and the breakpoints flanking the duplicated sequence were characterized at the nucleotide level by LR-PCR and Sanger sequencing (c.3114-811_3202-1756dup). A further characterization of this variant at the RNA level demonstrated the production of an abnormal transcript leading to the translation of a truncated protein (r.3114_3201dup; p.(Gly1068Glufs * 14)).

Until now, the association between *BRCA1/2* pathogenic variants and female/male breast cancer incidence, as well as ovarian and pancreatic cancer, has been widely reported. In addition to these genes, genetic alterations involving *PALB2* have been also described as implicated in these cancers. However, *PALB2* pathogenic mutations have not been extensively studied in patients with prostate cancer. In 2020, Yang et al. reported the results of a multicentric study conducted on 523 families and did not reveal any correlation between *PALB2* germline pathogenic variants and increased risk for prostate cancer [14]. In contrast, Wokolorczyk et al. have shown in 2021 that the two founder mutations of *PALB2* in the Polish population (c.509_510delGA and c.172_175delTTGT), which represent ~80% of all *PALB2* mutations, were commonly diagnosed within aggressive cancers of a high Gleason score (8–10) rather than middle Gleason score tumors (7) [15]. Other studies have established the occurrence of germline *PALB2* mutations in prostate cancer as well as the sensitivity of other *PALB2*-deficient tumor entities to Poly(ADP-ribose)polymerase (PARP) inhibition [16,17,18]. PARP inhibitors are oral agents that exert their activity through the concept of synthetic lethality [19]. At present, two PARP inhibitors are approved by the FDA for use in Castration-Resistant Prostate Cancer (*Opalarib* and *Rucaparib*) [20].

Usually, the genomic consequences of large duplications are difficult to infer without analyzing the RNA or determining the exact breakpoints. Thus, determining the genomic breakpoints of these large genomic rearrangements (LGRs) is essential to interpret their effect on genes and their correlation with phenotypes. In addition, analysis of genomic breakpoints can help characterize the molecular mechanisms behind these rearrangements. Two main mechanisms are involved: homologous (HR) and non-homologous recombination (NHR). The presence of highly homologous sequences in genomic breakpoints generally indicates HR, while the absence of sequence homology indicates a non-homologous mechanism [21]. To date, most of the reported pathogenic *PALB2* variants are spliced and nonsense/indel variants. Several pathogenic large duplications have been reported, such as exons 9–11 duplication [22] and exon 13 duplication [23]. Richardson et al. reported *PALB2* exon 11 duplication in his study about large duplications in breast cancer predisposition genes using a DNA breakpoint assay based on a custom NGS method. For *PALB2* EX11dup, two sets of breakpoints were identified in several families. One family had breakpoints in the *Alu*Ya5 and *Alu*Y element (5′: intron 10 and 3′: intron 11, respectively), while another proband had both breakpoints upstream of each of those breakpoints in the *Alu*Sz and *Alu*Sx element, respectively. In accordance with our study, we found the same reported first set of breakpoints (c.3114-811_3202-1756dup) that we have validated at both the DNA and RNA levels in an independent study. As previously reported in other studies that Alu-mediated HR is a frequent mechanism underlying *PALB2* large duplications [23,24].

In addition to the previous study conducted by Richardson et al. [24], this LGR with the same breakpoint was also identified in another unrelated family in our laboratory. The family had a female proband with a triple negative (TNG) breast cancer diagnosed with brain metastases at 36. The proband had, in her antecedent, a melanoma diagnosed at the age of 22 years (Figure S2). By integrating the genetic information and the family history, in addition to molecular arguments, we could conclude that this LGR is pathogenic and recurrent in the region.

Besides improvements of genetic diagnosis for these two types of cancer, these findings may additionally have therapeutic implications. There is strong evidence that (likely) pathogenic *PALB2* variants confer breast cancer risks that are considered as moderate to high. Therefore, multidisciplinary committees should advise a close surveillance and discuss the indication of a contralateral prophylactic mastectomy in these patients [25]. Finally, our patient was treated with a total mastectomy of the right breast followed by chemotherapy, adjuvant radiotherapy, and hormone therapy (*Tamoxifen*). The patient was not receptive to the contralateral prophylactic mastectomy.

Overall, we characterized, in a man with a synchronous prostate and breast cancer at both the DNA and RNA levels, a tandem and direct intragenic duplication in *PALB2*, which likely occurred by Alu-mediated homologous recombination. These findings expand the phenotypic spectrum of *PALB2*-associated cancer and may improve the mutation-based screening and genetic diagnosis of breast cancer.

## 4. Materials and Methods

### 4.1. Subjects and DNA Extraction

The patient, at 59 years old, addressed by department of clinical genetics of the university hospital of Lyon (Hospices civils de Lyon), Lyon, France.

Total genomic DNA was extracted from blood samples using the automated procedure implemented on the STARlet platform (Hamilton Company, Reno, NV, USA).

### 4.2. NGS Analysis and MLPA Confirmation

All subjects in this study were tested after giving informed consent after genetic counseling.

The DNA of the proband was processed by the commercial Hereditary Cancer Solution (HCS) kit (SOPHiA GENETICS, Saint-Sulpice, Switzerland) as described previously [26]. A total of 26 genes were analyzed using the NGS method (*ATM*, *APC*, *BARD1*, *BRCA1*, *BRCA2*, *BRIP1*, *CDH1*, *CHEK2*, *EPCAM* (large rearrangement only), *FAM175A*, *MLH1*, *MRE11A*, *MSH2*, *MSH6*, *MUTYH*, *NBN*, *PALB2*, *PIK3CA*, *PMS2*, *PTEN*, *RAD50*, *RAD51C*, *RAD51D*, *STK11*, *TP53*, and *XRCC2*). Duplication of *PALB2* exon 11 was confirmed by multiplex ligation-dependent probe amplification (MLPA) using the SALSA MLPA P260 *PALB2-RAD50-RAD51C-RAD51D* probe mix (MRC Holland, Amsterdam, The Netherlands) according to the manufacturer’s instructions.

### 4.3. Long-Range PCR and Breakpoint Determination

The characterization of the genomic breakpoint of the *PALB2* exon 11 duplication was based on the hypothesis that this LGR occurred in tandem and in direct orientation.

Since then, an LR-PCR has been performed using specific duplicating primers, in which the forward primer is located at the beginning intron 11 of *PALB2* and the reverse primer at the end of intron 10 (Figure 1A). Subsequently, to narrow the distance to the duplication breakpoint, a primer walking strategy was used with several forward primers located at intron 11 and reverse primers at intron 10. All PCR reactions were performed using Platinum™ SuperFi™ PCR Master Mix (Thermo Fisher Scientific, Invitrogen, Villebon sur Yvette, France) according to the manufacturer’s instructions. Amplified products were sequenced on a 3730 Genetic Analyzer using Big Dye Terminator Chemistry (Thermo Fisher Scientific, Waltham, MA, USA) according to the manufacturer’s recommendations. In each amplification, two normal samples were used to control PCR specificity. PCR conditions were denatured at 98 °C for 5 min for 35 cycles (denaturation 98 °C for 30 s, annealing 60 °C for 10 s, extension 72 °C for 4 min), followed by final extension at 72 °C for 5 min.

The duplication junction was amplified with a specific primer forward F-11 AGACCGCATCTTTCCCTAGC located in intron 11 and reverse R10-ATAAAGTTTGTTGGAATCACTTCCC located in intron 10, followed by direct sequencing to confirm the breakpoint. Sequencing reactions were performed with the BigDye Terminator v.3.1 cycle sequencing kit (Applied Biosystems, Waltham, MA, USA) on an ABI 3730XL sequencer according to the manufacturer’s instructions. The sequences were aligned against the wild-type *PALB2* nucleotide sequence (NM_024675; transcript ID).

### 4.4. RNA Analysis

RNA from the proband was extracted from the PAXgene Blood RNA Kit (PreAnalytiX, Qiagen, Valencia, CA, USA) and used for cDNA synthesis (Superscript III First-Strand Synthesis SuperMix, Invitrogen, Villebon sur Yvette, France). PCR was performed using a Platinum™ Taq DNA Polymerase (Thermo Fisher Scientific, Invitrogen, Villebon sur Yvette, France) and primers covering a region between exon 9 and 12 (forward: GTTAGTAGCAGTGGGACCCT-3′ and reverse: 5′-TCACAATGAGCTGAAACACA 3′) with the following reaction conditions: 95 °C for 4 min, initial denaturation, 14 cycles of 1 min at 95 °C, 1 min at 62 °C with an increase of 0.5 °C every PCR cycle, 2 min at 72 °C, 25 cycles of 1 min at 95 °C, 1 min at 55 °C, 2 min at 72 °C followed by 7 min at 72 °C for subsequent Sanger sequencing. Control RNA was extracted from HBC patients without the *PALB2* exon 11 duplication. The detailed protocol is available on request. The recommendations of the French Unicancer genetic Group were followed for the interpretation of the results [27].

## Data Availability

The data presented in this study are available on request from the corresponding author. The data are not publicly available due to restrictions of patient privacy.

## Supplementary Materials

The following are available online at https://www.mdpi.com/article/10.3390/ijms23020667/s1.
