# Supplementary material for: Identification and Characterization of an Exonic Duplication in PALB2 in a Man with Synchronous Breast and Prostate Cancer
Source: Int J Mol Sci. 2022 Jan 8;23(2):667. doi: 10.3390/ijms23020667 (PMC8775416; doi:10.3390/ijms23020667)
Supplement: Supplementary file 1 [file ijms-23-00667-s001.zip › ijms-1523117-supplementary.pdf]

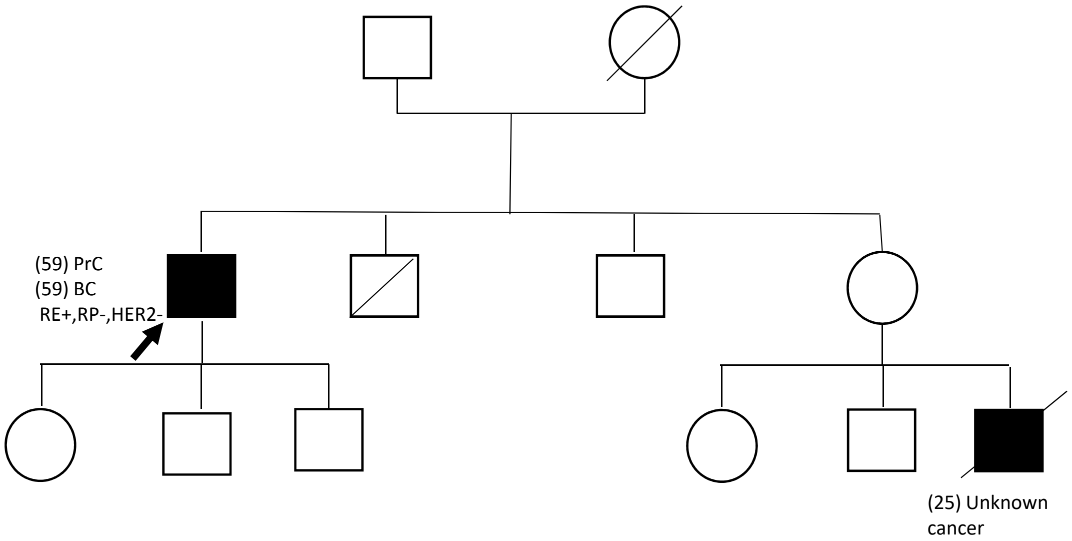

Figure S1 :

Patient pedigree. The patient (indicated with the arrow) is 59 -year-old man who was diagnosed a synchronous breast and prostate cancers , nephew was died after an unknown cancer at 25 (medical records were not available).

BC, breast cancer; PrC, prostate cancer; RE, estrogen receptor; RP, progesteron receptor;

Circle, female; square, male; filled symbols, individuals with cancer diagnosis; cross-hatched symbols, individuals already deceased.

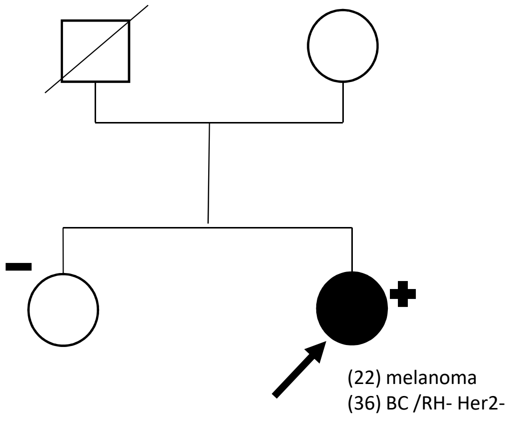

Figure S2 :
Patient pedigree of the second family harboring the mutation identified in our laboratory. The patient (indicated with the arrow) is a 36 -year-old woman who was diagnosed a triple negative breast cancer at 36. BC, breast cancer; RH, hormone receptors; (+), carrier of the PALB2 Ex11 dup; (-), non carrier of the mutation,
